# *Plasmodium falciparum,* vivax and malariae detection during the low transmission season in the hill tracts of Bangladesh

**DOI:** 10.1186/1475-2875-9-S2-P2

**Published:** 2010-10-20

**Authors:** Jasmin Akter, Sabeena Ahmed, Chai Prue, Wasif Khan, David Sack, Rashidul Haque, David Sullivan

**Affiliations:** 1 International Centre for Diarrhoeal Disease Research, Bangladesh

## 

A recent active cross sectional survey by a rapid diagnostic test (RDT) in 2007 showed crude malaria prevalence is 11% among the 1.5 million people most at risk for malaria in Bangladesh. An active randomized population malaria surveillance was initiated by JHMRI and ICDDR,B in Kuhalong union near hypoendemic Bandabarn in the Chittagong hill tracts of Bangladesh. The population of Kuhalong is 11,000 with approximately 2,000 households which were enumerated in a baseline census with a GIS mapping component. A demographic surveillance system and knowledge attitude and practice surveys have been obtained as part of the project to follow mosquito vectors by monthly trapping, host susceptibility factors and malariometrics. Here we present results from weekly malariometrics during the October to March low transmission season in the age groups of less than 5, 5 to 15 and greater than 15 years. Microscopy, RDT and real-time PCR was performed on active surveillance showing approximately 2% positive rates by microscopy or RDT in close to 500 individuals. A real-time PCR assay detected 6% prevalence. The sensitivity of the RT-PCR in the 96-well format was increased to 10-100 parasites per microliter with a glycogen/ acetate DNA precipitation at low speed tabletop centrifugation after column extraction. We detected *P. vivax* and *P. malariae* in less than 5% of the malaria positive patient samples by RT-PCR. All the *P. falciparum* isolates were chloroquine resistant PfCRT K76T genotype and atovaquone sensitive PfCYTb 268Y by fluorescent TAQman probe analysis. A reverse transcriptase real-time PCR assay from dried blood on filter papers was able to detect gametocytes. Studies on malaria seropositivity rates are in progress. In summary, a significant asymptomatic malaria PCR positive population exists. The role of this subpopulation in contributing to continuing transmission is being evaluated. Chloroquine resistance is fixed in this geographic area still responsive to artemisinin combination therapy.

